# Coatings in Decellularized Vascular Scaffolds for the Establishment of a Functional Endothelium: A Scoping Review of Vascular Graft Refinement

**DOI:** 10.3389/fcvm.2021.677588

**Published:** 2021-07-29

**Authors:** Jun Wei Heng, Muhammad Dain Yazid, Mohd Ramzisham Abdul Rahman, Nadiah Sulaiman

**Affiliations:** ^1^Centre for Tissue Engineering and Regenerative Medicine, Faculty of Medicine, Universiti Kebangsaan Malaysia, Kuala Lumpur, Malaysia; ^2^Department of Surgery, Hospital Canselor Tuanku Muhriz, Universiti Kebangsaan Malaysia, Kuala Lumpur, Malaysia

**Keywords:** decellularize, coating, endothelial, scaffold, treatment, small diameter, cardiovascular disease

## Abstract

Developments in tissue engineering techniques have allowed for the creation of biocompatible, non-immunogenic alternative vascular grafts through the decellularization of existing tissues. With an ever-growing number of patients requiring life-saving vascular bypass grafting surgeries, the production of functional small diameter decellularized vascular scaffolds has never been more important. However, current implementations of small diameter decellularized vascular grafts face numerous clinical challenges attributed to premature graft failure as a consequence of common failure mechanisms such as acute thrombogenesis and intimal hyperplasia resulting from insufficient endothelial coverage on the graft lumen. This review summarizes some of the surface modifying coating agents currently used to improve the re-endothelialization efficiency and endothelial cell persistence in decellularized vascular scaffolds that could be applied in producing a better patency small diameter vascular graft. A comprehensive search yielding 192 publications was conducted in the PubMed, Scopus, Web of Science, and Ovid electronic databases. Careful screening and removal of unrelated publications and duplicate entries resulted in a total of 16 publications, which were discussed in this review. Selected publications demonstrate that the utilization of surface coating agents can induce endothelial cell adhesion, migration, and proliferation therefore leads to increased re-endothelialization efficiency. Unfortunately, the large variance in methodologies complicates comparison of coating effects between studies. Thus far, coating decellularized tissue gave encouraging results. These developments in re-endothelialization could be incorporated in the fabrication of functional, off-the-shelf alternative small diameter vascular scaffolds.

## Introduction

Cardiovascular diseases (CVD) describe a variety of linked pathologies affecting the heart and blood vessels and are often associated with ischemic tissue damage predominantly as a consequence of severe arterial occlusion resulting from common underlying conditions such as atherosclerosis (1). Accountable for approximately 17.9 million deaths every year with projected annual mortalities rising to a staggering 23.3 million by 2030, CVDs represent a significant public health concern and is currently the leading cause of mortality and morbidity around the globe (2, 3). Despite these fatality rates, rapid identification and rectification of modifiable atherosclerotic risk factors through the employment of noninvasive treatment options such as dietary and lifestyle modifications (e.g., lipid control, smoking cessation) and pharmaceutical therapies (e.g., statins) can retard the occlusive process which greatly reduces the risk of premature CVD (4, 5). Patients experiencing severe arterial occlusion, as defined by the presence of >50% stenosis in the left coronary artery or >70% stenosis in a major coronary vessel (6), may opt for invasive vascular procedures such as percutaneous coronary intervention (PCI) or coronary artery bypass grafting (CABG) to achieve revascularization in affected arteries over extended periods of time (7). CABG has been associated with improved survival rates and reduced recurrence of major cardiovascular events and compared to PCI, particularly in patients with multivessel occlusions, however, making it the preferable treatment option over PCI (8–10).

The saphenous vein represents the current bypass conduit of choice for CABG procedures despite exhibiting poor long-term patency and high graft failure rates (~50% failure after 10 years) compared to autologous arterial grafts such as the internal mammary artery due to its ease of harvest with minimal complications (11, 12). Nevertheless, the use of autologous vessels for vascular bypass grafting comes with its own set of issues—vessel removal could cause damage at the extraction site and the extracted vessel may be of poor quality, thus preventing it from being used as a bypass conduit (13). Developments in tissue engineering techniques have allowed for the circumvention of these limitations, however, as the decellularization technique can be utilized to fabricate alternative biocompatible vascular scaffolds derived either from allogeneic or xenogeneic sources (14). These decellularized scaffolds can be used as conduits for vascular bypass, eliminating the need for autograft surgery.

The decellularization process involves striking a fine balance between the targeted removal of cellular and nuclear material contained in existing vascular tissues whilst minimizing damage to the extracellular matrix (ECM) constituents (14). This can be achieved *via* treatment of vascular tissues with chemical agents such as detergents and alcohols, biological agents such as enzymes and chelating agents, and physical methods including freeze-thawing, hydrostatic pressure, and non-thermal irreversible electroporation (15). Successful decellularization allows vascular scaffolds to retain their biomechanical properties whilst reducing their immunogenicity (13, 14, 16). Inadequate cellular depopulation could lead to immune-mediated damage which could result in graft failure. Overly aggressive decellularization is also unfavorable as it potentially results in the elimination or disruption of critical ECM components, which would adversely affect the structural integrity and mechanical properties of the decellularized tissue (17–19). Decellularized vascular scaffolds have exhibited their capability of supporting the adhesion and development of endothelial cells (EC) and smooth muscle cells (SMC) in multiple studies (20–22), making the prospect of producing a non-immunogenic tissue-engineered alternative small diameter vascular graft very promising.

A number of commercially available decellularized vascular grafts derived from bovine blood vessels and bovine ureters have been utilized in bypass surgeries in the past, but wide-scale implementation and utilization of these grafts as bypass conduits have not occurred to date due to their poor clinical outcomes post-implantation in regard to low patency and premature graft failure as a consequence acute thrombogenicity and intimal hyperplasia (13, 23). Multiple studies have implied that the absence of a luminal EC layer in decellularized vascular grafts is responsible for the development of thrombosis and ultimately premature graft failure upon *in vivo* implantation (13, 24–29). This occurs as the presence of a viable luminal endothelium aids to prevent exposed collagen from triggering the extrinsic blood coagulation pathway when coming into contact with peripheral blood, therefore an absence of ECs would result in thrombosis (29, 30). Intimal hyperplasia also represents a significant factor adversely affecting the long-term patency of decellularized scaffolds (27, 31, 32), in which a thickening of the neovascular neointima is observed. Although the exact mechanism responsible for the development of intimal hyperplasia is not fully understood, excessive vascular SMC migration and proliferation from the vessel media to the intima along with excessive ECM protein deposition have previously been suggested to be the cause (33). The presence of a compliance mismatch between the vascular graft and its adjacent native vessel, and the absence of a functional endothelium are some additional causes of intimal hyperplasia that have been identified (34–36). Nevertheless, the risks of the aforementioned conditions can be mitigated through careful donor species, site, and tissue processing and antigen removal methodology selection alongside timely re-endothelialization with functional ECs (37, 38).

All tissue-engineered vascular grafts (TEVGs), including decellularized scaffolds, will encounter a certain degree of re-endothelialization post-transplantation. Current identified mechanisms reveal that a majority of the re-endothelialization processes required for the generation of an effective and persistent endothelium occurs spontaneously during *in situ/in vivo* regeneration, but *in vitro* re-endothelialization involving luminal cell seeding and maturation prior to graft implantation remains the most commonly used technique to achieve re-endothelialization in TEVGs to date (39). The aforementioned *in situ* re-endothelialization processes include host intima ingrowth from the anastomotic regions predominantly occurring in TEVGs less than 2 cm in length (trans-anastomotic ingrowth) (40, 41), migration of capillary endothelium from adventitial granulation tissue onto the intimal surface (trans-mural capillary ingrowth) (42, 43), and deposition of circulating endothelial progenitor cells (EPCs), a small subset of CD34^+^ mononuclear cells capable of differentiating into an EC-like phenotype whilst expressing EC-specific markers, through recognition of proteins adsorbed on the surface of graft (fallout endothelialization) (39, 44, 45). Upon successful re-endothelialization and cellular repopulation, Henry et al. observed graft remodeling with an influx of MHC^+^ SMCs localized within the graft wall, and extensive matrix deposition of the ECM components collagen and elastin with a high percentage of cellular infiltration into graft (46). An increase in collagen and elastin deposition would be beneficial for the mechanical properties of a TEVG as collagen density has been shown to directly correlate with strength and stiffness whilst elastin imparts extensibility (47, 48).

Nevertheless, achieving and maintaining a healthy and functional endothelial lining has proven to be tricky. Depending on the length and diameter of the scaffold in question, *in situ* re-endothelialization mechanisms may or may not suffice for the generation of fully patent vascular grafts. Vascular grafts with an inner diameter size larger than 6 mm have demonstrated excellent patency (93%) after a period of 5 years post-implantation without the need for active *in vitro* re-endothelialization (49, 50), but these results cannot be extrapolated to shorter grafts with smaller inner diameters (<4 mm diameter, <5 cm length) as these grafts are extremely prone to acute thrombotic obstruction post-implantation; a study revealed that 5 of 6 rats implanted with non-endothelialized decellularized rat abdominal aortas succumbing to endothelial damage and acute thrombosis after 3 days with a final graft patency of 50% after 14 days, while 4 out of 6 rats remained alive after 14 days of implanting the reendothelialized graft, retaining a graft patency of 63% (51). Despite achieving confluent *in vitro* endothelialization prior to implantation of the reendothelialized grafts, graft patency and rat survival rates were significantly lower than that of the control (undecellularized rat abdominal aorta; 100% patency and survival). This occurrence likely resulted from exposure to high shear stresses from the pulsatile blood flow present in the circulatory system; previous studies have reported a maximal cell loss of 70% in EC-seeded grafts within minutes of exposure to flow (52, 53).

As such, enhancing the efficiency of the re-endothelialization process and improving EC retention post-implantation proves to be critical for the development of functional small diameter decellularized vascular scaffolds. One approach that can be utilized to achieve these goals is through the functionalization of the surface of decellularized scaffolds through the application and integration of specific proteins, such as growth factors, as bioactive surface coatings or modifications. This approach has previously been shown to result in grafts with improved patency with lower risks of thrombosis and intimal hyperplasia (32, 54–56). In the case of the previously explained study conducted by Hsia et al., sphingosine-1-phosphate (S1P), which has been shown to possess antithrombotic and pro-angiogenic properties (57), was utilized as the bioactive coating of choice (51). The results of their study demonstrated that rats implanted with re-endothelialized rat abdominal aortas coated with S1P exhibited 100% survival and patency rates as a result of increased EC migration and adhesion strength (51). This rise in EC migration could possibly be attributed to the pro-angiogenic properties of the S1P coating, as endothelial cells have a propensity to proliferate toward angiogenic stimuli (39). In principle, a combination of *in vitro* cell seeding in the presence of a bioactive coating appears to be a promising strategy to enhance *in situ* endothelial regeneration, which is ideal to achieve a persistent and functional endothelium. Thus, this article aims to systematically review existing literature on various examples of surface modifications used improve the efficiency and efficacy of the re-endothelialization process for the production of functional decellularized vascular scaffolds.

## Methods

This review was conducted in accordance with the guidelines stated in the Preferred Reporting Items for Systematic Reviews and Meta-Analyses (PRISMA) statement (58) to systematically assess publications in regard to the application and effects of coatings on the re-endothelialization of decellularized vascular scaffolds.

Electronic databases including PubMed, Scopus, Web of Science (WOS), and Ovid were utilized to acquire relevant primary studies, with results shown up to January 2021. The specific string used for all database searches was “((Decellularis^*^ OR Decellulariz^*^) AND (Arter^*^ OR Vein OR Vessel)) AND (coat^*^) AND (endotheliali^*^ OR populat^*^) NOT (review)” to ensure the inclusion of studies involving the coating of decellularized vascular scaffolds and cellular repopulation or re-endothelialization whilst excluding review articles. Search results were screened based on title and only publications regarding relevant primary studies written in the English language were included. Publications qualifying through the preliminary title screening were subjected to abstract screening, and only articles with abstracts that illustrated the use of decellularized tissues or organs coated with bioactive molecules that have been shown to positively affect endothelial cell or endothelial progenitor cell recruitment/adherence/attachment/coverage or the re-endothelialization/cellular repopulation process were shortlisted for inclusion. Publications utilizing modifications that aim to improve the feasibility of decellularized grafts as potential cardiovascular therapeutics but do not affect the re-endothelialization process such as those enhancing graft mechanical properties or reducing intimal hyperplasia/thrombosis without enhancing endothelial coverage were deemed ineligible as the scope of this review is to enhance the re-endothelialization process. All eligibility assessments were conducted by two reviewers to reduce author/selection bias in the article selection process, and disagreements regarding the eligibility of included publications and/or data were resolved through discussion between reviewers. Publications meeting any of the following exclusion criteria were also eliminated from further review: non-English language articles, conference abstracts, news articles, letters, editorials, case studies, and review articles.

Duplicate entries were removed from the inclusion group, and a data extraction table (Table 1) was generated to summarize the following information from included publications: author, tissue decellularized, coating used, and findings.

**Table 1:** Summary of publications selected from electronic database searching.

**No**.	**Authors**	**Tissue decellularized**	**Coating used**	**Findings**
1	Hussein et al. (59)	Porcine liver (right lateral lobe)	Heparin-gelatin	Heparin-gelatin precoating of the decellularized scaffolds increased cell recruitment and strength of attachment of EA.hy926 ECs onto vessel walls.
2	López-Ruiz et al. (60)	Porcine carotid artery	Poly(ethylmethacrylate-codiethylaminoethylacrylate) (8g7)	8g7 is a biocompatible and readily synthesizable polymer coating that enhances attachment of ECs promoting endothelium regeneration while reducing platelet attachment and improving biomechanical properties of decellularized vessels.
3	Bär et al. (61)	Porcine small intestine (BioVaM)	CCN1	CCN1 coating resulted in enhanced quantity of cells within the decellularized BioVaM structure, but increased adherence of cells does not necessarily mean improved overall function.CCN1 improves angio- and vasculogenic properties *via* integrins as the presence of the RGD integrin inhibitor prevented CCN1 action.
4	Walawalkar S, Almelkar S. (62)	Bovine aorta	Fibrin glue (FG)	FG coating supports cellular DNA synthesis, rapid endothelialization, and proliferation.Presence of vWF and leptin indicate that FG-coating on the basal lamina does not result in differentiation of EC to a different lineage.Fibrin glue sealant can be used to support re-endothelialization of large diameter vessels.
5	Marinval et al. (63)	Porcine heart valves	Fucoidan-VEGF	Coating decellularized heart valves with fucoidan-VEGF improves HUVEC adhesion, cell density, and cell viability in both static and dynamic cultures.Coated samples were not immunogenic and exhibited improved re-endothelialization and were able to confer enhanced protection against thrombosis without increasing calcification.
6	Kim et al. (64)	Rat liver	Anti-CD31 ssDNA aptamer	Anti-CD31 aptamer coating of the vascular lumen of decellularized liver scaffolds promotes re-endothelialization, thereby leading to formation of perfusable vascular networks with enhanced viability and functionality *via* activation of integrin-Akt signaling cascades.
7	Böer et al. (65)	Equine carotid artery	CCN1	CCN1 coating facilitated attachment of ECs to the matrix and induced neomedia formation and neovascularization. Inflammatory reactions were reduced, and immunologic tolerance was induced.Thus, CCN1 coating improved remodeling and biocompatibility of decellularized xenogenous matrices significantly.
8	Tsai et al. (66)	Murine descending aorta	VEGF	Local VEGF application enhances EC-phenotype differentiation in the vessel grafts.VEGF promoted re-endothelialization and reduced neointimal lesions in decellularized vessel grafts, possibly due to VEGF's ability to induce precursor differentiation.
9	Musilkova et al. (67)	Human pericardium	**Crosslink:** Glutaraldehyde, Genipin **Coating:** Fibrin, Heparin, Fibronectin	Modifying decellularized pericardium by means of genipin crosslinking combined with fibrin-based coating offers a potential means for stimulating the *in situ* endothelialization of non-autologous pericardial grafts.
10	Iijima et al. (68)	Murine aorta	VEGF conjugated to temperature-sensitive aliphatic polyester hydrogel (HG-VEGF)	VEGF stimulates EC adhesion to ligands of the basement membrane, promotes formation of a functional neo-endothelium, and stabilizes the neoendothelium in front of shear forces generated by the blood stream.Although coated grafts showed higher percentage of functional endothelium, the rate of neo-intimal hyperplasia was also increased.Coated grafts showed strong augmentation of neo-intimal hyperplasia between the 4 and 8th week *in vivo*, leading to a significantly increased intima-to-media ratio.
11	Lee et al. (69)	Canine inferior vena cava and jugular vein	**Polydopamine (pDA) coating** Modified with: •RGD •YIGSR	pDA-mediated peptide immobilization enhanced adhesion, metabolic activity, and endothelial differentiation of hEPCs, all of which are factors likely to promote endothelium formation on the luminal surface of matrices.
12	Assmann et al. (70)	Rat aorta	Fibronectin	Fibronectin coating induced medial graft repopulation without inflammatory reactions or adverse gene expressions, indicating the feasibility and potential of this strategy for the improvement of current clinically applied bioprostheses.
				Despite accelerating the endothelialization of cardiovascular implants, fibronectin may not represent the optimal coating agent since it promotes intimal hyperplasia.
13	Conklin et al. (71)	Porcine carotid artery	Heparin pre-treatment + bFGF coating	The bFGF coating increases the proliferation rate of both HMECs and CEPCs *in vitro* and may therefore help expedite the endothelialization process *in vivo*.
14	Flameng et al. (72)	Ovine aortic valve	Fibronectin + stromal cell-derived factor 1α (FN/SDF-1α)	Long-term results of this study can be explained by the homing of the appropriate progenitor cells, but it is more than probable that the milieu provided by the FN/SDF-1a coating instigates a cellular reaction leading to faster healing and better controlled tissue deposition and inflammatory reaction.
15	Wan et al. (73)	Rat pancreas	GRGDSPC peptide	GRGDSPC can effectively bind to the decellularized pancreatic scaffold and promote the proliferation of HUVECs, which may be related to the upregulation of integrins αvβ3, α5β1, and αIIbβ3, thus, promoting EC recruitment and functional endothelialization.
16	Leuning et al. (74)	Human kidneyRat kidney	VEGF Angiopoietin-1 (Ang-1)	Reconstituting growth factors is critical for cell adherence and survival and is a key step to facilitate maximal endothelial coverage.Re-endothelialization of the kidney vasculature can be conducted with controlled arteriovenous delivery and culture of hIPSC-derived ECs.

## Results

### Search Results

**Figure 1:**
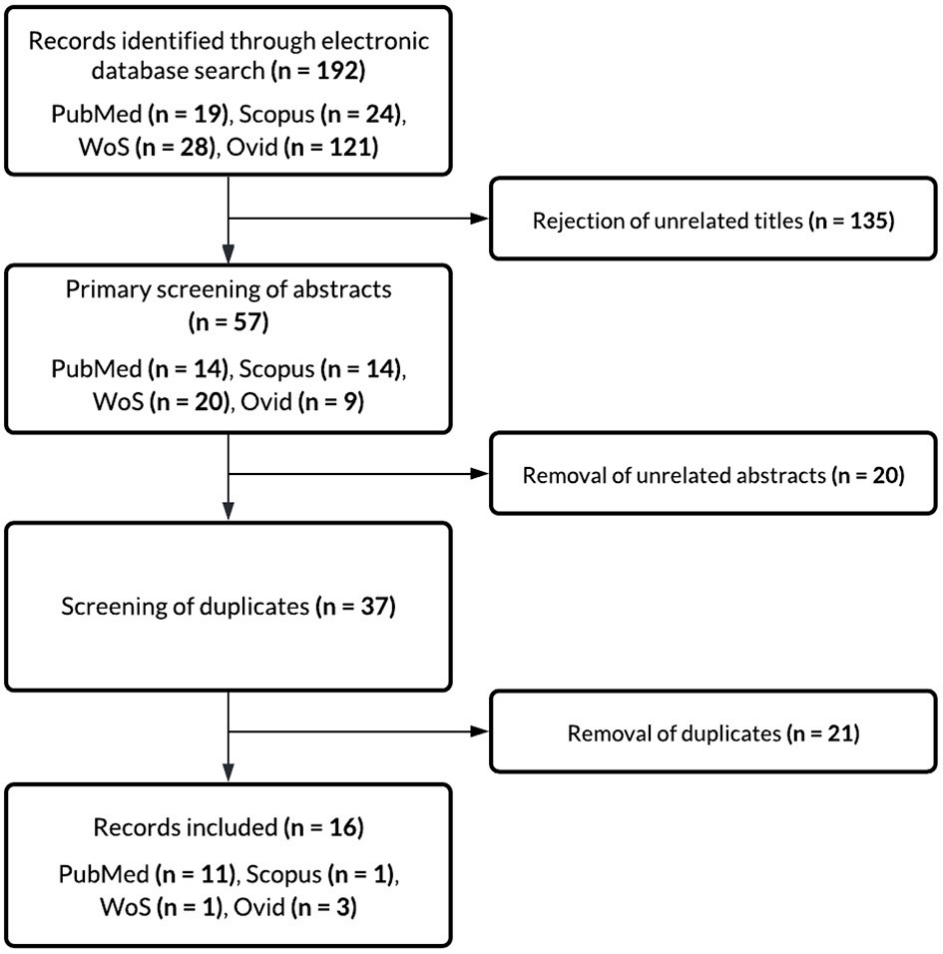
Flow chart depicting selection criteria.

Primary database search yielded a total of 192 results, of which 19 articles were derived from PubMed, 24 articles from Scopus, 28 articles from WoS, and 121 articles from Ovid. Independent screening of the initial results was conducted by two reviewers in accordance with the inclusion and exclusion criteria to reduce bias in the selection process, followed by a joint discussion which resulted in the unanimous decision to eliminate 135 unrelated articles and include 57 articles: 14 from PubMed, 14 from Scopus, 20 from WoS, and 9 from Ovid. Abstracts were carefully screened, and 20 articles were removed as they did not match the inclusion criteria, leaving a total of 37 articles in the inclusion group. Duplicate entries were screened for, and 21 articles were removed, resulting in a final tally of 16 publications, of which 11 were from PubMed, one from Scopus, one from WoS, and three from Ovid. A flow chart depicting the selection process is shown in Figure 1.

### Study Characteristics

Decellularized biological scaffolds were utilized in all selected studies. However, the type of tissue decellularized and its origin differed. Decellularized scaffolds were modified through the addition of a coating agent prior to re-endothelialization in all included publications, but the coating agent utilized varied between each study. A summary of included publications is provided in Table 1.

## Discussion

Sixteen publications were selected from a pool of 192 results as they utilized different decellularized vascular scaffolds coated with various substances to improve re-endothelialization. Information regarding the types of decellularized vascular scaffolds and coatings utilized in the studies are summarized in Table 1. Effects of the coating agents on the re-endothelialization of decellularized vascular grafts are further discussed.

### Heparin

The sulphated polysaccharide heparin has been well-documented as a potent, endothelial cell-binding anticoagulant agent. Heparin impedes the development of thrombosis *via* inhibition of specific serine proteinases involved in the blood coagulation cascade through the potentiation of antithrombin III (75). Hussein et al. (59) established that the coating of the decellularized porcine liver right lobe with heparin-gelatin, significantly increases attachment of EA.hy926 endothelial cells on vascular surfaces whilst reducing endothelial cell migration into the parenchyma. 3-(4,5-dimethylthiazol-2-yl)-2,5-diphenyl tetrazolium bromide (MTT) assay results showed a 3.8-fold increase in cell attachment on heparin-gelatin coated samples compared to uncoated control samples. Heparin and gelatin coated samples yielded a smaller 2.1-fold and 2.5-fold increase, respectively. Increased EC attachment was observed in heparin-gelatin coated samples likely due to an increase presence of VEGF and bFGF, as these growth factors exhibit high affinity for heparin, and ECs have been shown to express receptors for these growth factors (59, 76–78).

Musilkova et al. (67) used a different approach for their study, where decellularized human pericardium (DP) was strongly or weakly crosslinked with glutaraldehyde and/or genipin prior to coating with a fibrin mesh modified with heparin, fibronectin, heparin and fibronectin, or unmodified. Genipin acts as a natural, low-toxic crosslinking agent with prior experiments demonstrating its ability to stabilize decellularized scaffolds with minimal cytotoxicity and immunogenicity as compared to glutaraldehyde-crosslinked decellularized scaffolds (79–82). Results from Musilkova et al.'s study indicated that glutaraldehyde crosslinking, irrespective of crosslinking strength, resulted in a reduction of seeded human umbilical vein endothelial cells (HUVEC) metabolic activity (MTA), while genipin crosslinking increased MTA across all DP samples. Highest MTA was produced in HUVECs seeded onto DP weakly crosslinked with genipin and coated with fibrin and fibronectin. MTA in samples coated with heparin-modified fibrin mesh and heparin and fibronectin-modified fibrin mesh were lower as compared to other treatments. Despite exhibiting lower MTA values, the anticoagulatory and anti-inflammatory effects of heparin-fibrin may be beneficial in inhibiting thrombosis prior to re-endothelialization (67, 83).

Heparin-treated decellularized porcine carotid arteries were coated with bFGF in Conklin et al.'s study (71) to analyze the impact of bFGF on the proliferation of human microvascular endothelial cells (HMEC) and canine endothelial progenitor cells (CEPC). The decellularized carotid arteries were pre-treated with heparin to enhance adhesion of bFGF as it is a heparin-binding growth factor that has been previously shown to enhance EC migration and proliferation, angiogenesis, and re-endothelialization (84–87). Experimental results shown that the coating improves *in vitro* proliferation rate of both HMECs and CEPCs, with bFGF-coated samples seeing a 2.4-fold increase in HMEC and 2.3-fold rise in CEPC cell quantities compared to uncoated samples after 4 days and 2 days, respectively. HMECs seeded on coated scaffolds were also found to be more resistant than uncoated samples when cultured under shear stresses (71).

### VEGF

VEGF was also utilized in a number of studies as it functions as a powerful angiogenic and mitogenic agent that is capable of inducing EC migration and proliferation to accelerate the vascularization process when bound to its specific VEGF receptors (88–91). A study conducted using decellularized murine descending aorta coated with VEGF established that incorporation of the growth factor facilitated the re-endothelialization process and led to decreased development of neointimal lesions in the murine aortic graft (66). This occurrence is presumably a result of VEGF's capacity to trigger differentiation of lesional progenitor cells toward an endothelial lineage (66), as evidenced by the development of endothelial-specific markers (eNOS, vWF, CD31, CD144, and VEGFR 1/2) on lesional progenitor cells cultured *in vitro* in the presence of VEGF (66). The authors also demonstrated that these *in vitro* results were extrapolatable to *in vivo* subjects, as local application of VEGF onto the implanted vascular graft decreased neointimal development and promoted EC localization to surface of the graft (66).

Iijima et al. (68) explored the impacts of coating decellularized murine aorta with VEGF conjugated to a temperature-sensitive aliphatic polyester hydrogel (HG-VEGF) which allows for a steady and sustained release of the growth factor (92). They concluded that the sustained VEGF exposure enhanced EC adhesion to the basement membrane and promoted the formation of a functional endothelial layer, with HG-VEGF coated samples presenting 64.8 ± 7.6% EC coverage on their luminal surface 4-weeks post-treatment, while uncoated samples presented 40.4 ± 8.3% EC coverage. Medial repopulation was also increased in coated vessels, with the absolute cell count of coated samples at 4 weeks and 8 weeks being 7.3 ± 5.9 cells, and 22.1 ± 13.0 cells, respectively. Uncoated vessels had 0.80 ± 1.2 cells at 4 weeks and 3.2 ± 3.6 cells at 8 weeks. Despite the promising increase in re-endothelialization, use of the coating eventually resulted in neointimal hyperplasia, which led to a significantly increased intima-to-media ratio (68).

Marinval et al. (63) proposed the modification of decellularized porcine heart valve scaffolds *via* a multi-layer application of the brown algae-derived sulphated polysaccharide, fucoidan, and VEGF (63, 93). Multiple studies have shown that fucoidan promotes the adhesion, migration, and proliferation of endothelial cells, and has antithrombotic properties similar to that of heparin but carries a lower hemorrhagic risk than the former (93–96). The authors disclosed that the coating led to an improvement in HUVEC adhesion accompanied by enhanced cell density and viability on the decellularized valvular scaffold in both static and dynamic cultures. Visual examination of HUVEC re-endothelialization in static culture conditions showed endothelial cells presenting as a highly connective homogenous monolayer, with a larger number of adherent, living cells present 6-h post-treatment with fucoidan/VEGF compared to untreated samples. Similar evaluations conducted on HUVECs cultured under perfusion revealed ECs aligning toward the direction of perfusion with greater endothelial cell adhesion in coated samples. Cell viability under perfusion was also significantly enhanced, with coated samples showing 4,549 ± 325 viable cells per field, while uncoated samples showed 3,343 ± 292 cells per field. Fucoidan/VEGF coating can be used to enhance re-endothelialization whilst reducing the risk of thrombosis (63).

Human and rat kidneys were decellularized by Leuning and co-workers (74), and the effects of VEGF and Ang-1 coating on decellularized kidney scaffolds were investigated. Growth factor loading was determined to be an essential measure for maximal endothelial cell adherence, survival, and coverage, as samples coated with VEGF and Ang-1 exhibited increased EC adherence and viability. Human induced pluripotent stem cell-derived endothelial cells (hIPS-ECs) were also seeded onto the coated samples, and similar results were produced; VEGF and Ang-1 coating resulted in enhanced EC adherence and viability. hIPS-ECs were also seeded on decellularized human kidney, and the authors reported enhanced hIPS-EC proliferation in VEGF + Ang-1 coated samples with the potential to scale the re-endothelialization process throughout the entire kidney. Minimal vascular obstructions were also observed in coated samples, highlighting the importance of growth factor reconstitution for the re-endothelialization of decellularized kidney scaffolds (74).

### Cellular Communication Network Factor 1 (CCN1)

CCN1 is a secreted surface-associated pro-angiogenic matricellular protein that is recognized to mediate cell adhesion and migration, and cell proliferation through integrin interaction and induction of growth factor-associated DNA synthesis respectively (97–100). CCN1 has also recently been implicated to be capable of inducing the recruitment and localization of circulating CD34^+^ progenitor cells to the endothelial layer, contributing to the regeneration of the endothelium due to their ability to differentiate into mature ECs (101–103). The recruited EPCs may also positively influence angiogenesis and neovascularization through the paracrine secretion of pro-angiogenic cytokines (104, 105).

The study conducted by Bär et al. (61) utilized human cord blood-derived endothelial cells (hCBEC) to repopulate decellularized porcine small intestines with preserved pedicles (BioVaM) coated with CCN1. hCBEC attachment experiments were conducted on plates coated with gelatin or CCN1-enriched gelatin. Results showed significantly improved hCBEC adhesion and retention when seeded on CCN1-enriched gelatin-coated plates compared to hCBEC seeded on plates coated with only gelatin. When hCBECs were seeded on the decellularized scaffolds, enhanced re-endothelialization efficiency was observed in CCN1-coated BioVaMs compared to their uncoated counterparts (84 ± 9% cell retention vs. 47 ± 4% cell retention). DNA content analyses conducted 12 h post-re-endothelialization revealed significantly higher DNA content in coated samples (37 ± 2 μg/g) compared to uncoated samples (11 ± 3 μg/g), clearly showcasing the effects of CCN1 coating on decellularized porcine small intestine scaffolds (61).

A similar study (65) conducted using decellularized equine carotid arteries coated with CCN1 observed that the CCN1 coating not only facilitates circulating endothelial cell attachment, but also induces endothelial and smooth muscle cell proliferation, neomedia formation, organized neovascularization, reduced local inflammatory reactions, and induced immunological tolerance which collectively enhances biocompatibility of decellularized vascular grafts significantly (65).

### Fibronectin

Fibronectin (FN) represents a major ECM constituent that has the capacity to induce endothelial cell adhesion, migration, and differentiation through conjugation or adhesion with biomaterial surfaces (106). Therefore, Assmann et al. (70) and Flameng et al. (72) conducted studies utilizing FN as a coating for their decellularized vascular scaffolds to mimic what has been done *in vitro* to assess FN applicability in 3D.

Assmann et al. (70) investigated the effects of FN on the autologous *in vivo* re-endothelialization of decellularized murine aortic conduits and concluded that FN greatly enhanced EC adhesion capacity and decellularized graft biocompatibility, resulting in accelerated re-endothelialization. Examination of the luminal surface 8-weeks post-treatment showed 89.9 ± 5.45% repopulation on the luminal surface of the coated samples, while uncoated samples displayed 73.6 ± 13.14% re-endothelialization. Immunofluorescence also revealed that the cells present in the luminal zone stained positive for vWF, confirming the presence of endothelial cells. Unfortunately, additional findings also suggest that FN exacerbated the development of hyperplastic neointima. Thus, FN may not represent the optimal coating agent for decellularized vascular grafts (70).

Flameng et al. (72) coated decellularized ovine aortic valves with fibronectin and stromal cell-derived factor 1α (FN/SDF-1α) and noticed that the coating substantially improved re-endothelialization performance on coated decellularized samples, displaying values comparable to that of native cryopreserved aortic grafts, which exhibited 39 ± 8% re-endothelialization at the leaflet region and 37 ± 5% at the wall region of the aortic graft 5 months post-implantation in Lovenaar sheep. In contrast, uncoated decellularized samples only demonstrated 10–15% re-endothelialization within the same timeframe (72).

### Biomimetic Peptides

RGD (arginine-glycine-aspartic acid) represents an extensively studied biomimetic peptide that has been widely utilized as a surface modifier for biomaterials. RGD has been shown to be capable of stimulating cell adhesion, cell migration, and cell proliferation through specific recognition and interaction with integrins (107, 108). However, RGD peptides exhibit low biological activity innately, but this issue is easily counteracted *via* the introduction of chemical modifications to RGD to form biologically active peptides GRGDS and GRGDSPC (109). Research conducted by Wan et al. (73) modified decellularized murine pancreas with the GRGDSPC peptide to stabilize HUVECs on the scaffold. Immunofluorescent staining revealed that both GRGDSPC-conjugated samples and uncoated samples expressed Ki67 and CD31, suggesting that HUVECs effectively adhered and proliferated in groups, however, the levels of these markers were substantially higher in GRGDSPC-conjugated samples, indicating greater biocompatibility for growth and proliferation of HUVECs. The study concludes that the GRGDSPC peptide successfully binds to the pancreatic scaffold facilitating HUVEC proliferation and functional endothelialization, presumably through enhanced expression of integrins αvβ3, α5β1, and αIIβ3 (73).

In their study, Lee et al. (69) incorporated a mussel-inspired polydopamine (pDA) coating for use on a decellularized canine vein matrix consisting of the inferior vena cava and jugular vein (DVM). pDA-coated decellularized vein matrices (pDA-DVMs) were then conjugated with the RGD and YIGSR peptides to produce (CGGRGD)-pDA-DVMs and (CGGYIGSR)-pDA-DVMs, respectively. Human cord blood-derived endothelial precursor cells (hCB-EPCs) and human embryonic stem cell-derived endothelial precursor cells (hESC-EPCs) were seeded onto pDA-coated DVMs (pDA-DVM) to analyze the effects of conjugated and unconjugated pDA coatings on the efficiency of re-endothelialization. Increased metabolic activity in hCB-EPCs seeded onto (CGGYIGSR)-pDA-DVMs were observed in comparison to uncoated DVMs and pDA-DVMs. qRT-PCR suggested enhanced precursor cell differentiation into endothelial cells on the peptide-modified DVMs, as indicated by an increased expression of endothelial specific markers, with the largest increase seen in cells seeded on (CGGRGD)-pDA-DVMs. Adhesion of hEPCs on peptide modified DVMs was also improved compared to uncoated control samples. With these results, Lee et al. confirmed that the modified PDA coating positively impacted EC adhesion and metabolic activity whilst inducing the differentiation of hEPCs into an endothelial lineage. All these factors contribute to increased re-endothelialization efficiency in decellularized vascular scaffolds (69).

### Other Coating Agents

López-Ruiz et al. (60) demonstrated improved re-endothelialization with decreased thrombosis risk in decellularized porcine carotid arteries coated with the polymer poly(ethylmethacrylate-co-diethylaminoethylacrylate) (8g7). 8g7 has been previously described as a novel biocompatible polymer that carries the ability to facilitate and improve EC adhesion and viability (110). DAPI staining revealed cellular repopulation occurring in a similar manner in both 8g7-coated and uncoated samples, but with a significant higher number of visible cells attached to the coated sample. Platelet adhesion testing on 8g7-coated coverslips revealed reduced platelet adhesion under increasing shear stresses in a flow system. Additionally, ECs grown on coated coverslips exhibited increased angiogenic properties as evidenced by the formation of capillary-like tubes after 4 h. Biomechanical testing of the samples showed a substantial difference in the vessel burst pressure between native, non-decellularized arteries and 8g7-coated decellularized arteries (1,330 ± 135 mbar vs. 1,153 ± 138 mbar). Tensile strength of coated samples, however, was comparable to native arteries, while uncoated decellularized arteries showed a much lower maximum load (60).

Fibrin glue (FG) is an essential biological adhesive generated from the activation of fibrinogen by thrombin and is essential in the blood coagulation cascade (111). This material was previously observed by Almelkar and colleagues to support angiogenesis, endothelial cell adhesion, migration, and proliferation (112). In their succeeding study, decellularized bovine aorta was coated with FG composed of a 1:1 ratio of fibrinogen and thrombin (62). Sheep external jugular vein endothelial cells (SEJVEC) cultured on coated and uncoated samples revealed that SEJVECs seeded onto FG-coated samples achieved full confluency in 5 days, with visual examinations showing that the seeded cells assumed a flat morphology with an expansion of filopodia, comparable to endothelial cells found in physiological conditions. Cell viability was also unaffected by the coating. In contrast, SEJVECs seeded onto uncoated samples only achieved 70% confluence after 10 days while adopting a cobblestone morphology. Immunocytochemistry revealed presence of vWF and lectin on FG-coated samples which was absent on uncoated samples. These results indicate that fibrin glue is a non-toxic coating that can be used to greatly enhance the re-endothelialization of large diameter decellularized vascular scaffolds (62).

Kim et al. (64) proposed using an anti-CD31 aptamer coating to promote re-endothelialization in decellularized murine liver scaffolds. Nucleic acid aptamers are short sequences of synthesized single-stranded oligonucleotides that have low immunogenicity and high binding affinity for specific proteins, and thus, are often considered alternatives to antibodies. Compared to antibodies, however, aptamers are often easily adjusted and producible in massive amounts at a relatively low cost with limited risk of variability and contamination (113–115). Kim et al. determined that anti-CD31 aptamer coating of decellularized liver scaffolds facilitates re-endothelialization. These contribute to the development of vascular networks that can support perfusion with increased functionality and viability through the potentiation of integrin-Akt signaling cascades (64). ECs seeded onto anti-CD31 aptamer coated grafts exhibited significantly higher cell attachment compared to uncoated grafts or anti-CD31 antibody coated grafts. Attached ECs were also more resistant to shear stresses and were less likely to detach, with aptamer-coated grafts retaining 57.84 ± 2.9% cell adhesion under shear stress and uncoated grafts retaining 21.87 ± 1.2%. HUVEC endothelial coverage on aptamer-coated grafts were also significantly higher after 7 days in culture, at 76.10 ± 3.54% coverage compared to HUVECs on uncoated grafts which had 35.22 ± 7.74% endothelial coverage (64).

Although the re-endothelialization of decellularized small dimeter vascular grafts in the context of cardiovascular diseases remain the priority for this review, the studies included utilized both decellularized vascular grafts and decellularized tissues due to the limited number of studies conducted involving bioactive coatings or surface modifications and decellularized small diameter vascular grafts. Nevertheless, the results obtained from these studies remain valuable as the core goal is to improve reendothelialization efficiency and endothelial cell retention in decellularized scaffolds whilst contending against shear stresses post-implantation. As explained previously, exposure of decellularized grafts lacking a functional endothelium to the blood stream increases the risk of acute thrombogenesis and premature graft failure, and as such, improving the efficiency the reendothelialization process is critical to minimize contact between these surfaces to reduce the chances of the aforementioned risks. The coatings discussed in the included studies generally function to enhance *in situ* fallout endothelialization and transmural capillary ingrowth by increasing adhesion, migration, and proliferation while promoting angiogenesis in endothelial and/or endothelial progenitor cells. VEGF, Fibronectin, and CCN1/RGD peptides represent the most utilized coatings. A similarity between these molecules is that they share is their ability to interact with various subtypes of the cell surface receptor integrin present on endothelial cells and endothelial progenitor cells, forming the basis for their effects on promoting endothelialization (65, 116, 117).

To elaborate using VEGF as an example, the interaction between VEGF-A and its specific tyrosine kinase receptor VEGFR2 results in the autophosphorylation of the receptor and thus, the activation of the receptor. The association between VEGFR2 and integrin α_v_β_3_ expressed on circulating ECs and EPCs leads to the phosphorylation of the β_3_ subunit of the integrin, resulting in its activation which in turn upregulates VEGF expression, induces cell migration and activates the mitogen-activated protein kinase (MAPK) pathway, which has been shown to be involved in progenitor cell proliferation, differentiation, and survival (117–120), among many other crucial downstream processes (121–123). Fibronectin and CCN1 are both also similarly able to interact with different integrin subtypes, i.e., FN and CCN1 with integrin α_v_β_3_, FN with α_5_β_1_, and CCN1 with α_6_β_1_ (124, 125) to carry out the aforementioned functions which helps improve *in situ* re-endothelialization mechanisms for the production of functional decellularized vascular grafts. Some studies described in this review resorted to coating with a combination of bioactive materials or pre-treating their decellularized grafts with different proteins prior to application of the main coating agent, such as can be seen in Hussein et al.'s (59) study combining heparin with gelatin, and Marinval et al.'s (63) study incorporated fucoidan with VEGF. This is a promising approach that could enhance the effectiveness of existing coating materials, as the benefits the secondary material, such as the antithrombogenic and anticoagulant properties of fucoidan and the myriad of integrin binding sites present on gelatin, could be garnered to produce a more effective coating. The potential complications such as cell toxicity that could arise from combining these coatings has to be taken into consideration, however. Nevertheless, this could be an avenue for future studies aiming to improve the quality of re-endothelialization in decellularized vascular scaffolds.

## Conclusion

**Figure 2:**
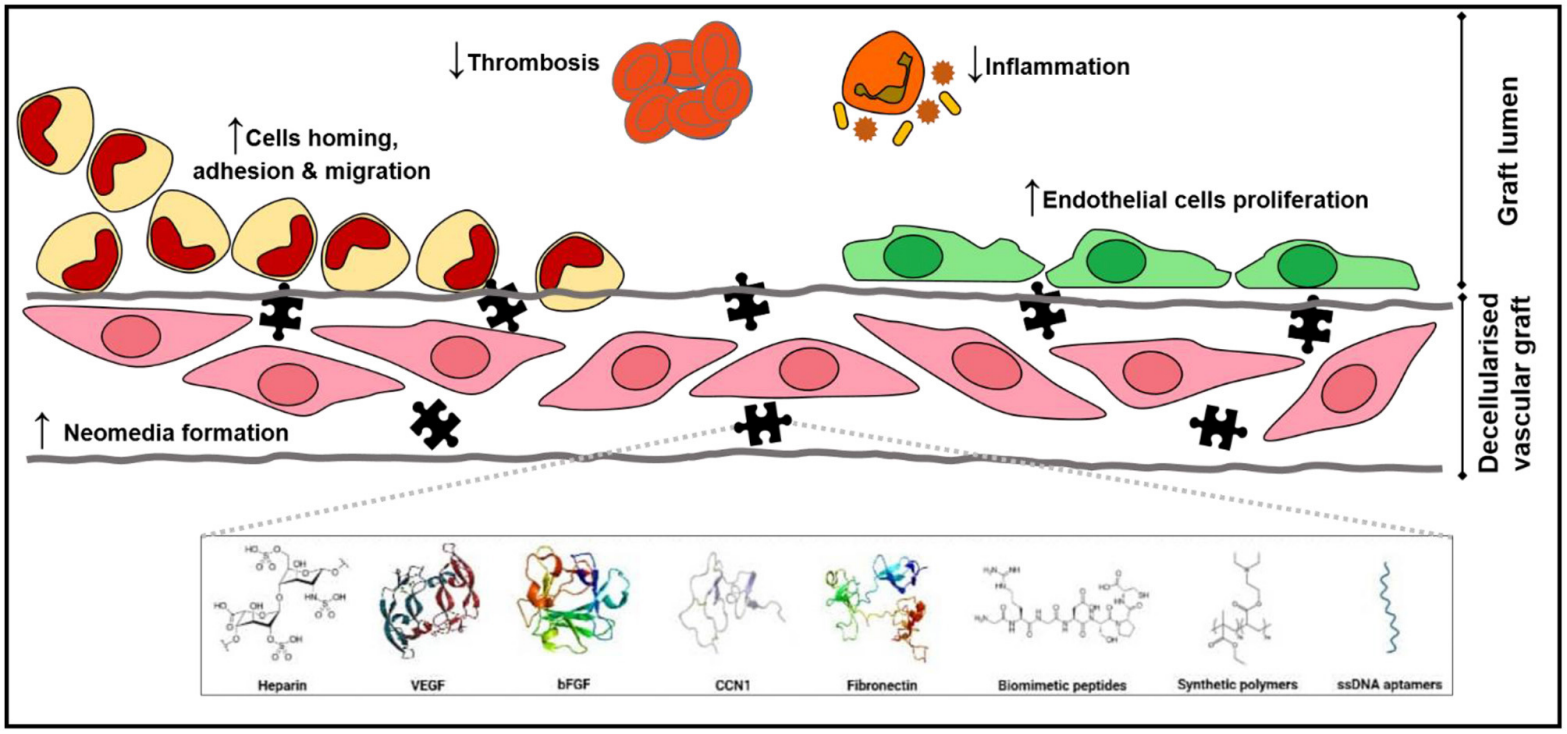
The missing puzzle piece. Coatings of decellularized vascular graft present a myriad of positive effects that promote cell adhesion, migration, and proliferation both on the luminal surface, enhancing reendothelialization, and in the decellularized vascular graft, establishing a neomedia that consequently strengthens mechanical properties of the graft. Coatings also halt thrombosis and reduce inflammatory reactions.

Multiple different coating agents and their effects on re-endothelialization have been discussed in this review, and the use of coating agents are capable of inducing EC adhesion, migration, and proliferation resulting in enhanced re-endothelialization efficiency. A schematic representation of the various positive effect of these coatings on decellularized vascular grafts are illustrated in Figure 2. However, the variation in testing methodologies and types of decellularized tissues complicates the comparison of coating effects across the studies. This is illustrated when comparing the studies conducted by Iijima et al. (68) and Assmann et al. (70) with other studies employing the same coating agents, as both studies reported significant neointimal hyperplasia while the same did not occur in other studies. Thus, a more standardized analysis or criteria should be established to enable more robust comparison across studies. Furthermore, future studies could compare the performance of different coating agents to ascertain the optimal reagent required to produce the highest re-endothelialization efficiency. In addition, the feasibility of large-scale implementation of these coating methodologies as well as the long-term potential of coated scaffolds is also yet to be determined. Nevertheless, these findings are promising and suggest that the creation of fully functional non-immunogenic off-the-shelf tissue engineered vascular graft alternatives could be feasible sooner than later with additional research.

## Data Availability Statement

The original contributions generated for this study are included in the article/supplementary material, further inquiries can be directed to the corresponding author/s.

## Author Contributions

NS, MY, and MA laid out the broad initial research question for this systematic review. JH, NS, and MY designed the search and refine the research question. JH performed article selection and screening, data collection and extraction, manuscript writing, and data analysis. NS oversees, review and verify articles selection, data collection, and analysis. MY and MA performed final proofreading of the manuscript. All authors contributed to the article and approved the submitted version.

## Conflict of Interest

The authors declare that the research was conducted in the absence of any commercial or financial relationships that could be construed as a potential conflict of interest.

## Publisher's Note

All claims expressed in this article are solely those of the authors and do not necessarily represent those of their affiliated organizations, or those of the publisher, the editors and the reviewers. Any product that may be evaluated in this article, or claim that may be made by its manufacturer, is not guaranteed or endorsed by the publisher.
